# Pain in the heart, hair on the head: Pericarditis as an unanticipated risk of low-dose oral minoxidil

**DOI:** 10.1016/j.jdcr.2026.07.056

**Published:** 2026-08-04

**Authors:** Isabella Zappi, Archie Spindler, Derek Maas, Hooman Yaghoobzadeh, Kristen I. Lo Sicco, Jerry Shapiro

**Affiliations:** a The Ronald O. Perelman Department of Dermatology, NYU Grossman School of Medicine, New York, New York; b University of Rochester School of Medicine, Rochester, New York; c Department of Internal Medicine, Weill Cornell Medicine, New York, New York

**Keywords:** androgenetic alopecia, cardiovascular side effects, low-dose oral minoxidil, oral minoxidil, pericarditis

## Introduction

Low-dose oral minoxidil (LDOM) has emerged as an off-label first-line therapy for alopecia.^1^ Higher antihypertensive doses of minoxidil are limited by rare but serious cardiovascular risks, including pleural effusion, pericardial effusion, and pericarditis.^2^ Nonetheless, studies investigating systemic adverse events of LDOM have demonstrated that it is generally well tolerated.^2^ Seldom cases of oral minoxidil-associated pericarditis have been reported, mostly occurring at antihypertensive doses (10-40 mg) or in patients with underlying cardiac or renal disease.^3^ Two notable cases of pericarditis have been associated with LDOM (<5 mg) for androgenetic alopecia (AGA),^4^^,^^5^ with additional reports of pericardial and pleural effusions.^6^ Although uncommon, LDOM-associated pericarditis warrants prudent attention. Herein, we present a case of LDOM-associated pericarditis in a young, otherwise healthy man and review reported cases in the literature.

## Case report

**Fig 1:**
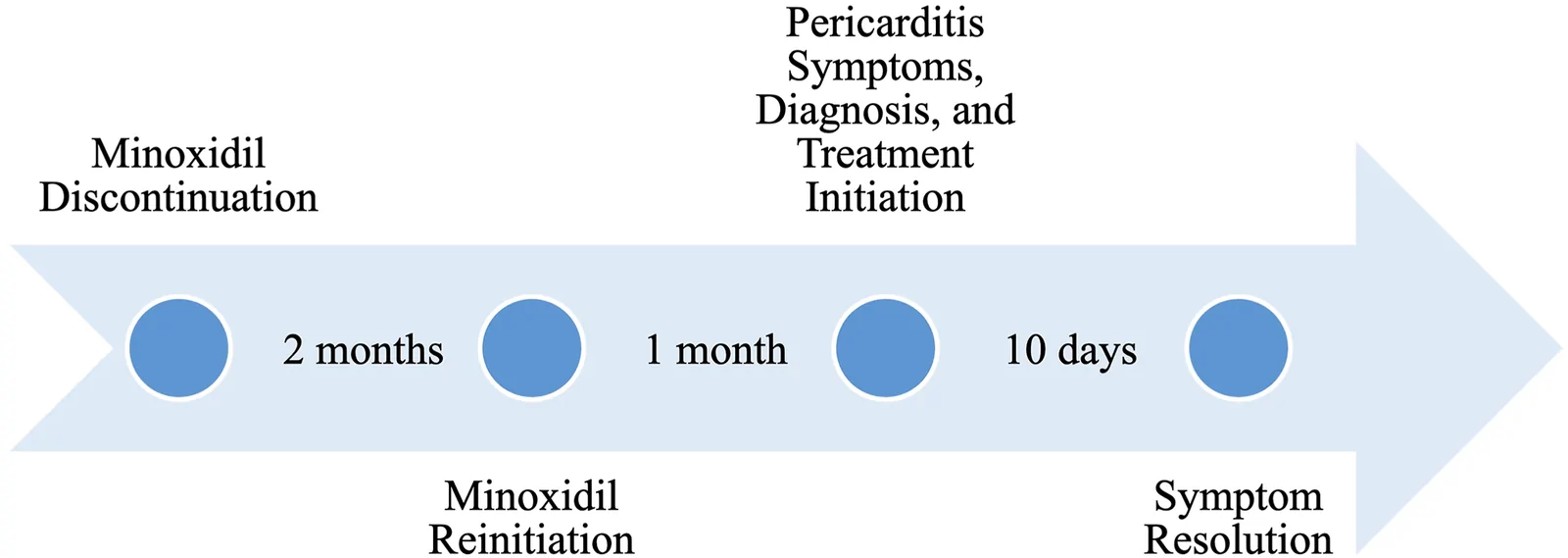
Low-dose oral minoxidil therapy was restarted after a 2-month discontinuation. Pericarditis symptoms developed 1 month later at which point he was subsequently diagnosed and treated with resolution of symptoms within 10 days.

A 33-year-old otherwise healthy man with AGA and no cardiac or renal history presented to his primary care physician, a cardiologist, with pleuritic, positional chest pain and shortness of breath 1 month after restarting LDOM (5 mg daily) (Fig 1). He previously tolerated the same dose of LDOM for over 2 years without adverse effects. Following a 4-month discontinuation, he independently restarted LDOM at 5 mg without consulting his dermatologist or cardiologist. Electrocardiogram demonstrated diffuse ST elevations, while echocardiogram revealed a normal ejection fraction with no evidence of a pericardial effusion. Chest x-ray exhibited no pleural effusion. The patient denied viral symptoms (fever, chills, fatigue) or recent vaccinations. Autoimmune workup with a rheumatologist, including antinuclear antibody, a full antibody panel, and Lyme titers, was negative except for an elevated rheumatoid factor (rheumatoid factor; 28; reference <14 IU/mL) (Table I). Inflammatory markers revealed an elevated high-sensitivity C-reactive protein (13.8, week 1; 23.5, week 2; 16.6, week 3; reference 0.0-3.0 mg/L) and erythrocyte sedimentation rate (6, week 1; 22, week 2; 15, week 3; 0.0-22.0 mm/h) (Table II). D-Dimer was negative. White blood cell count was within normal limits (9; reference 3.8-10.8 × 10^3^/μL). Pericarditis was clinically diagnosed by his cardiologist. LDOM was discontinued and colchicine and non-steroidal anti-inflammatory drugs were initiated with symptom resolution within 10 days.

**Table I:** Autoimmune workup

Test	Result	Reference range and comments
Antinuclear antibody (ANA) by immunofluorescence (IFA)	Negative	Negative
Rheumatoid factor (RF)	28	<14 IU/mL
Anticyclic citrullinated peptide antibody (Anti-CCP)	<16	Negative: <20 Weak positive: 20-39 Moderate positive: 40-59 Strong positive: >59
Antidouble stranded DNA antibody (dsDNA)	<1	≤4 negative 5-9 intermediate ≥10 positive
Anti-Smith antibody (Anti-Sm)	<1.0 negative	<1.0 negative
Anti-ribonucleoprotein antibody (Anti-RNP)	<1.0 negative	<1.0 negative
Anti-Ro/SSA antibody	<1.0 negative	<1.0 negative
Anti-La/SSB antibody	<1.0 negative	<1.0 negative
Anti-Scl-70 (anti-topoisomerase I) antibody	<1.0 negative	<1.0 negative
Anti-centromere B antibody	<1.0 negative	<1.0 negative
Anti-RNA polymerase III antibody	<20	<20
Anti-histone antibody IgG	<1.0	<1.0 negative, 1.0-1.5 weak positive, 1.6-2.5 moderate positive, >2.5 strong positive
Angiotensin converting enzyme	26	9-67 U/L
Complement, C3	148	82-185 mg/dL
Complement, C4	28	15-53 mg/dL
HLA B high resolution typing	B-1 B∗14:02 B-2 B∗49:01	
Lyme total antibodies, IgG and IgM western blot	Lyme disease Ab(IgG), Blot: NEGATIVE Lyme disease Ab(IgM), Blot: NEGATIVE 18 KD (IgG) Band: REACTIVE 23 KD (IgG) Band: NON-REACTIVE 28 KD (IgG) Band: NON-REACTIVE 30 KD (IgG) Band: NON-REACTIVE 39 KD (IgG) Band: NON-REACTIVE 41 KD (IgG) Band: NON-REACTIVE 45 KD (IgG) Band: NON-REACTIVE 58 KD (IgG) Band: NON-REACTIVE 66 KD (IgG) Band: NON-REACTIVE 93 KD (IgG) Band: NON-REACTIVE 23 KD (IgM) Band: NON-REACTIVE 39 KD (IgM) Band: NON-REACTIVE 41 KD (IgM) Band: NON-REACTIVE	Lyme disease Ab(IgG), Blot: NEGATIVE Lyme disease Ab(IgM), Blot: NEGATIVE

Urinalysis		
Color	Yellow	Yellow
Appearance	Clear	Clear
Specific gravity	1.003	1.001-1.035
pH	7.0	5.0-8.0
Glucose	Negative	Negative
Bilirubin	Negative	Negative
Ketones	Negative	Negative
Occult Blood	Negative	Negative
Protein	Negative	Negative
Nitrite	Negative	Negative
Leukocyte esterase	Negative	Negative
White blood cell (WBC)	None seen	≤5/HPF
Red blood cell (RBC)	None seen	≤2/HPF
Squamous epithelial cells	None seen	≤5/HPF
Bacteria	None seen	None seen/HPF
Hyaline casts	None seen	None seen/LPF
Protein and creatinine, urine		
Creatinine, random urine	19	20-320 mg/dL
Protein, total, random urine	<4	5-25 mg/dL

**Table II:** Inflammatory marker and complete cell count workup

	Baseline (6 mo prior)	Wk 1	Wk 2	Wk 3	Reference range
High-sensitivity C-reactive protein	1	13.8	23.5	16.6	0.0-3.0 mg/L
Erythrocyte sedimentation rate	6	6	22	15	0.0-20.0 mm/h
D-dimer	-	0.26	0.44	-	<0.50 mcg/mL
White blood cell (WBC)	-	-	9	-	3.8-10.8 ×10^3^
Red blood cell (RBC)	-	-	5.05	-	3.80-6.50 ×10^3^
Hemoglobin (HGB)	-	-	14.7	-	13.2-17.1 g/dL
Hematocrit (HCT)	-	-	43.6	-	35.0% to 55.0%
Mean corpuscular volume (MCV)	-	-	86	-	80-100 um
Mean corpuscular hemoglobin (MCH)	-	-	29.1	-	26.0-34.0 pg
Mean corpuscular hemoglobin concentration (MCHC)	-	-	33.8	-	31.0-37.0 g/dL
Red cell distribution width (RDW)	-	-	13.5	-	10.0% to 20.0%
Platelet (PLT)	-	-	242	-	140-400 ×10^3^
Mean platelet volume (MPV)	-	-	8.9	-	6.0-10.0 um
Neutrophil %	-	-	74.7	-	38.0% to 80.0%
Lymphocyte %	-	-	17.3	-	15.0% to 49.0%
Monocyte %	-	-	6.9	-	0.0% to 13.0%
Neutrophil #	-	-	6.7	-	1.5-7.8 ×10^3^
Lymphocyte #	-	-	1.6	-	0.8-3.9 ×10^3^
Monocyte #	-	-	0.6	-	0.2-1.0 ×10^3^
Eosinophil %	-	-	0.8	-	0.00% to 8.00%
Eosinophil #	-	-	0.1	-	0.0-0.6
Basophil %	-	-	0.3	-	0.0% to 2.0%
Basophil #	-	-	0.03	-	0.00-0.20

The patient presented to the hair specialist dermatology clinic for AGA follow-up within 1 week of his initial cardiology visit, reporting increased shedding over the previous months following initial and current LDOM discontinuation. HairMetrix trichometric measurements demonstrated decreased terminal hair density and stable-to-worsened caliber in the frontal and vertex scalp compared to 1 year prior. Global scalp photography revealed diffuse thinning, particularly in the frontal and bitemporal regions. Trichoscopy revealed miniaturization and hair shaft diameter variability without black dots, broken hairs, or exclamation point hairs. Given the rapid, diffuse nature of hair loss, a 4 mm scalp punch biopsy was performed to rule out diffuse alopecia areata. Histopathology exhibited no evidence of scarring or inflammation, and negative Verhoeff-Van Gieson and Periodic Acid–Schiff with Diastase stains, findings consistent with AGA. Despite the patient’s desire to restart LDOM, dermatology and cardiology jointly advised against reinitiation. He instead initiated oral finasteride 1 mg daily and platelet-rich plasma scalp injections for AGA management.

## Discussion

### Differential diagnosis

• Following multidisciplinary discussion among dermatology, cardiology, and rheumatology, LDOM-associated pericarditis was considered the most likely diagnosis.
  ○ Temporal association with LDOM reinitiation
  ○ Largely negative autoimmune workup
• Minoxidil-induced systemic lupus erythematosus was considered but deemed unlikely despite prior reports^7^
  ○ Absence of anti-histone antibodies
  ○ Negative antinuclear antibody
  ○ No clinical features of systemic lupus erythematosus
  ○ Did not meet systemic lupus erythematosus diagnostic criteria
• Rheumatoid arthritis-associated pericarditis was considered unlikely.
  ○ Low-positive rheumatoid factor
  ○ No clinical evidence of rheumatoid arthritis
  ○ Failed to meet rheumatoid arthritis diagnostic criteria
• Viral pericarditis was considered less likely.
  ○ Absence of viral symptoms
  ○ Normal white blood cell count

### Alopecia: Diagnosis and therapeutic dilemma

• The patient’s increased hair shedding may reflect transient shedding following LDOM discontinuation and reinitiation, a well-documented side effect of LDOM.^8^
• Alternatively, stress-induced telogen effluvium may have further unmasked underlying AGA.
• Nonetheless, this adverse event limits therapeutic options, necessitating consideration of alternative oral or adjunctive treatments.

### Literature review

• An in-depth PubMed, Google Scholar, and Scopus search using terms “minoxidil pericarditis” and “oral minoxidil pericarditis” identified 2 additional LDOM-associated pericarditis cases (Table III)

**Table III:** Reported cases of low-dose oral minoxidil-associated pericarditis

Age/sex	Dose	Medical history	Diagnosis	Outcomes	Reference
53, Male	5 mg daily	Well-controlled diabetes, no renal or heart failure	Pericarditis, recurrent pericardial effusion	Resolution of symptoms and effusions upon discontinuation of minoxidil	Abdulhai et al (2024)^5^
52, Male	2.5 mg daily	Idiopathic pericarditis complicated by pericardial effusion (5 y prior)	Pericarditis, peripheral edema	Resolution of symptoms upon discontinuation of minoxidil	Bentivegna et al (2022)^4^
• Bentivegna et al.
  ○ Healthy 52-year-old man
  ○ Remote history of idiopathic pericarditis complicated by pleural effusion
  ○ Developed pericarditis and peripheral edema after 2 weeks of LDOM (2.5 mg daily) (Table I)^4^
• Abdulhai et al.
  ○ 53-year-old man
  ○ Well-controlled diabetes without underlying renal or heart disease
  ○ Developed pleural effusion followed by pericarditis 20 days after initiating LDOM (5 mg daily)^5^
• In both cases, infectious and autoimmune workups were unrevealing and symptoms resolved upon LDOM discontinuation without hospital admission or diuretic therapy.
• Although exceedingly rare, these reports highlight a potential association between LDOM and pericarditis.

### Clinical implications and takeaways

• Despite LDOM’s favorable safety profile, current studies may be underpowered to detect rare complications such as pericarditis.^8^
• This case underscores the potential cardiovascular risks of LDOM, including pericarditis, even in young, healthy individuals without pre-existing cardiac or renal disease.
• Prior tolerance to LDOM may not preclude side effects upon re-exposure.
• Lustig et al similarly described pericarditis in a patient after restarting antihypertensive-dose minoxidil (30 mg daily) following a brief discontinuation and years of uncomplicated use.^9^
• While our patient restarted LDOM at 5 mg without gradual titration—raising questions about whether higher starting doses contribute towards risk—pericarditis has also occurred at a lower dose of 2.5 mg.^4^
• This case reinforces the importance of dermatologist supervision when resuming LDOM, as pericarditis may develop rapidly and should warrant cardiac evaluation.
• These findings additionally emphasize the need for caution with direct-to-consumer telemedicine pharmacies that may prescribe LDOM with limited monitoring and follow-up.
• Clinicians should remain vigilant for pericarditis symptoms and counsel patients regarding the possible rare but serious cardiac complications.

## Conflicts of interest

Dr Lo Sicco is a prior investigator for Pfizer and Regen Lab; consultant for Pfizer, Lilly, Aquis, Priovant, Veradermics, and Ro; and board member for the Scarring Alopecia Foundation and American Hair Research Society. Dr Shapiro is an investigator for Pfizer and a consultant for Pfizer, Lilly, Replicel Life Science, Regen Lab, Thirty Madison, DS Laboratories, Aclaris Therapeutics, Incyte, and Almirall. Dr Yaghoobzadeh, and Authors Zappi, Spindler, and Maas have no conflicts of interest to declare.

## References

[1] Randolph M., Tosti A. (2021). Oral minoxidil treatment for hair loss: a review of efficacy and safety. J Am Acad Dermatol.

[2] Vañó-Galván S., Pirmez R., Hermosa-Gelbard A. (2021). Safety of low-dose oral minoxidil for hair loss: a multicenter study of 1404 patients. J Am Acad Dermatol.

[3] Yousaf H., Sharma A., Sbitli T. (2024). Minoxidil-induced acute pleuro-pericarditis with pleuro-pericardial effusion. J Am Coll Cardiol.

[4] Bentivegna K., Zhou A.E., Adalsteinsson J.A., Sloan B. (2022). Letter in reply: pericarditis and peripheral edema in a healthy man on low-dose oral minoxidil therapy. JAAD Case Rep.

[5] Abdulhai F., Parizher G., Zmaili M. (2024). Minoxidil-related pericarditis. JACC Case Rep.

[6] Dlova N.C., Jacobs T., Singh S. (2022). Pericardial, pleural effusion and anasarca: a rare complication of low-dose oral minoxidil for hair loss. JAAD Case Rep.

[7] Tunkel A.R., Shuman M., Popkin M., Seth R., Hoffman B. (1987). Minoxidil-induced systemic lupus erythematosus. Arch Intern Med.

[8] Akiska Y.M., Mirmirani P., Roseborough I. (2025). Low-dose oral minoxidil initiation for patients with hair loss: an international modified Delphi consensus statement. JAMA Dermatol.

[9] Lustig S., Pitlik S.D., Garty M., Rosenfeld J.B. (1985). Pericarditis after minoxidil reinstitution. Drug Intell Clin Pharm.

